# Evaluation of hair regrowth after minoxidil and dutasteride tattooing in men with androgenetic alopecia

**DOI:** 10.1016/j.jdin.2023.04.011

**Published:** 2023-05-20

**Authors:** Sara D. Ragi, Soha Ghanian, Nicole Rogers, Danielle M. Peterson, Luke S. Johnson, Carlos G. Wambier

**Affiliations:** a Department of Dermatology, The Warren Alpert Medical School of Brown University, Rhode Island Hospital, Providence, Rhode Island; b Hair Restoration of the South, Metairie, Louisiana; c Department of Dermatology, Yale School of Medicine, New Haven, Connecticut; d Department of Dermatology, University of Utah, Salt Lake City, Utah

**Keywords:** alopecia, androgenetic alopecia, antiandrogens, baldness, drug delivery, dutasteride, hair loss, male pattern hair loss, microneedling, minoxidil, minoxidil sulfate, tattoo machine

*To the Editor:* Topical minoxidil and oral 5-α-reductase inhibitors have been the usual care for male androgenetic alopecia (AGA) for decades. Such drugs have been adopted through intralesional injections for the treatment of AGA.^1^^,^^2^ Drug delivery with tattoo equipment^3^^,^^4^ has also been adopted with such medications to the balding scalp.^5^ The aim of this study was to evaluate the clinical outcome of 3 monthly sessions of minoxidil-dutasteride tattooing (MDT).

We conducted a retrospective analysis of patients who underwent MDT from 2016 to 2020 because of a lack of response to topical minoxidil and oral 5-α-reductase inhibitors for >6 months. We included all male patients who had received 3 monthly sessions. Out of 73 patients with at least 1 session, a total of 15 men were included. Deidentified pooled photographs (baseline and 4 months of follow-up) were randomized for top-quadrant Severity of Alopecia Tool (SALT) blinded scoring by 3 evaluators via an online survey. The primary outcome was defined as achieving >10% top scalp area regrowth (TSAR), calculated based on the difference in the top-quadrant SALT scores. The secondary outcomes included response rate per presence of vellus hair and scalp photoaging at the baseline. The patients gave consent for their photographs and medical information to be published in print and online and with the understanding that this information may be publicly available.

After cleaning the scalp with 70% isopropanol, a rotatory tattoo machine (Cheyenne, MT.DERM) set at 120 Hz using a 27-needle cartridge (3240 perforations/s) at 1.5-mm needle exposure was used to deliver sterile-compounded 0.5% minoxidil sulfate (1 mL) and 0.1% dutasteride (1 mL) (Pineda). The patients were authorized to continue oral 5-α-reductase inhibitors and instructed to resume topical minoxidil after a week after the procedure.

The median age was 49 years (range, 24-69 years), 9 patients (60%) exhibited signs of scalp photoaging, 8 (53%) had a high density of vellus hair, 1 (7%) previously underwent scalp microblading to conceal AGA, and 1 (7%) underwent hair transplant. The baseline median top SALT was 60% (interquartile range, 38%-78%) and 40% (interquartile range, 19%-67%) at 4 months (Wilcoxon signed-rank test, *P* < .001).

Eight patients (53%) achieved a mean TSAR of >10%, and 4 (27%) achieved a mean TSAR of >20% (Fig 1). Overall, the median TSAR was 10% (interquartile range, 5%-20%) (Supplementary Fig 1, available via Mendeley at https://data.mendeley.com/datasets/6r865wbgc7).

**Fig 1:**
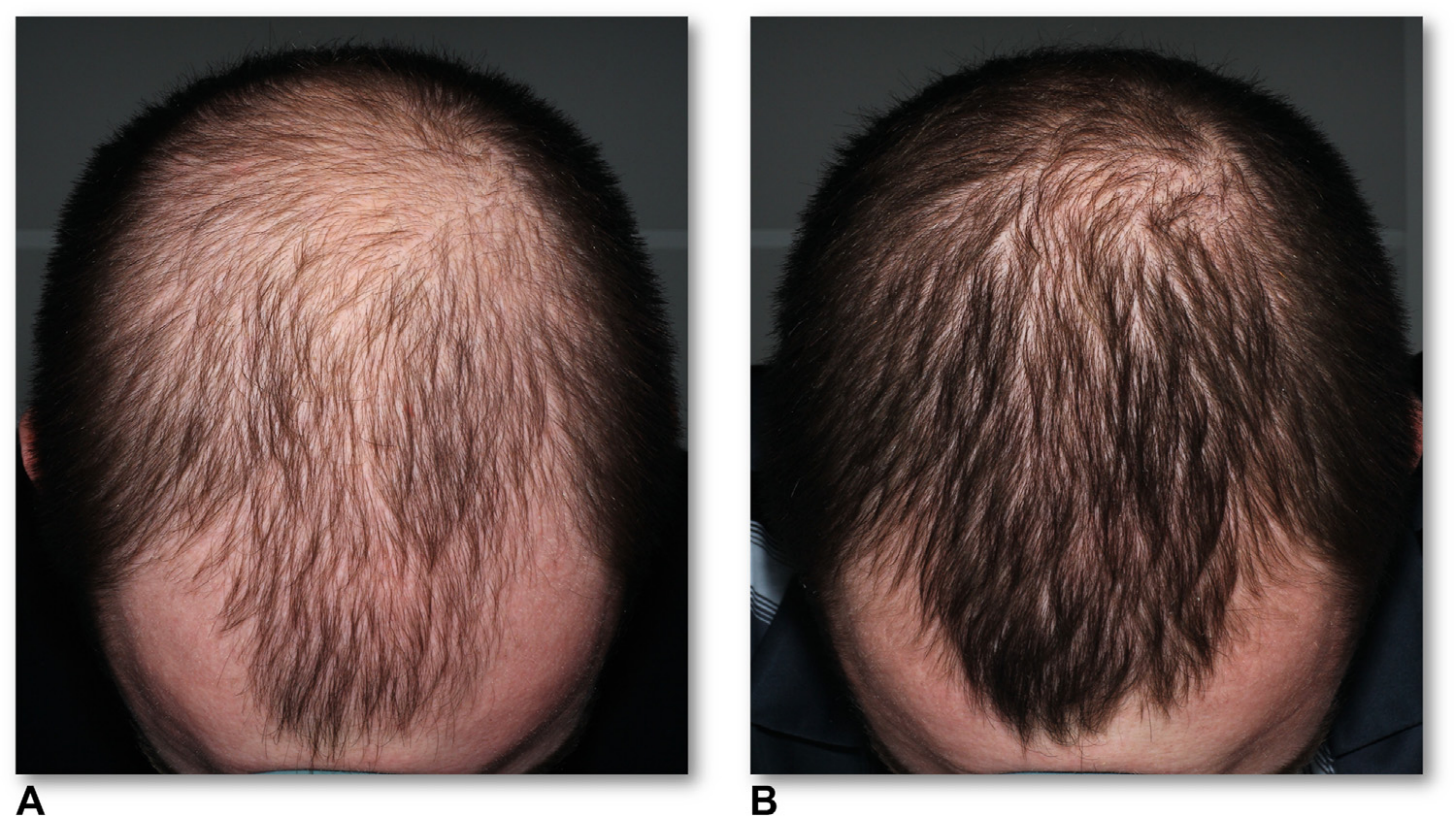
Androgenetic alopecia. Photographs with the same hair length. Illustrative standardized cross-polarized overhead images of the top quadrant utilizing Reveal Imager (Canfield Scientific) for top-quadrant Severity of Alopecia Tool scoring by evaluators. (**A**) Before, the top Severity of Alopecia Tool score was 53%. (**B**) After 4 months, the top Severity of Alopecia Tool score was 28%. A successful 25% top scalp area regrowth was observed after 3 sessions of minoxidil and dutasteride drug delivery through tattooing.

In patients with vellus hair at the baseline, 63% of the evaluations achieved >10% TSAR compared with 22% in those with no vellus hair (Fisher exact test, *P* = .014). In patients with scalp photoaging at the baseline, 26% achieved >10% TSAR compared with 78% in those with no scalp photoaging (*P* = .001). All patients reported scalp desquamation for 2 to 3 days in the first week after MDT. Infection, scarring, or worsened alopecia did not develop in any patient. Procedural pain was managed with topical anesthetics or neural blocks.

Most patients experienced significant hair growth, as measured based on TSAR, which was derived from the top-quadrant SALT scores using top standardized photography only (Fig 1). Histology or trichoscopy evaluations were not performed. A lack of response to MDT may be explained by a combination of factors, including low vellus hair density and scalp photoaging (such as pigmentation and solar lentigines) at the baseline.

Our findings provide preliminary evidence of the use of MDT for male AGA and useful information for future prospective double-blinded, randomized, placebo-controlled trials of drug delivery.

## Conflicts of interest

Dr Wambier has served as an adviser for Applied Biology, Chemistry Rx, Daniel Alain, and Young Pharmaceuticals and as an investigator for Concert Pharmaceuticals, Incyte, Eli Lilly, Pfizer, Sun Pharma, and UCB. Dr Peterson has served as investigator for Concert Pharmaceuticals, Incyte, Eli Lilly, Pfizer, and AnaptysBio. The other authors have no potential conflict of interest to disclose.

## References

[1] Villarreal-Villarreal C.D., Boland-Rodriguez E., Rodríguez-León S., Le Voti F., Vano-Galvan S., Sinclair R.D. (2022). Dutasteride intralesional microinjections in combination with oral minoxidil vs. oral minoxidil monotherapy in men with androgenetic alopecia: a retrospective analysis of 105 patients. J Eur Acad Dermatol Venereol.

[2] Uzel B.P., Takano G.H., Chartuni J.C. (2021). Intradermal injections with 0.5% minoxidil for the treatment of female androgenetic alopecia: a randomized, placebo-controlled trial. Dermatol Ther.

[3] Ghanian S., Wambier C.G. (2021). Response to “microneedling with autologous platelet-rich plasma versus microneedling with topical insulin in the treatment of postacne atrophic scars: a simultaneous split-face comparative study”. J Am Acad Dermatol.

[4] Arbache S., Godoy C. (2013). Microinfusion of drugs into the skin with tattoo equipment. Surg Cosmet Dermatol.

[5] Contin L.A. (2016). Male androgenetic alopecia treated with microneedling alone or associated with injectable minoxidil by microinfusion of drugs into the skin. Surg Cosmet Dermatol.

